# Transcriptome analysis identifies novel responses and potential regulatory genes involved in seasonal dormancy transitions of leafy spurge (Euphorbia esula L.)

**DOI:** 10.1186/1471-2164-9-536

**Published:** 2008-11-12

**Authors:** David P Horvath, Wun S Chao, Jeffrey C Suttle, Jyothi Thimmapuram, James V Anderson

**Affiliations:** 1 Biosciences Research Laboratory, USDA-Agricultural Research Service, Fargo ND, USA; 2 Northern Crop Science Laboratory, USDA-Agricultural Research Service, Fargo ND, USA; 3 WM Keck Center for Comparative and Functional Genomics, University of Illinois, Urbana IL, USA

## Abstract

**Background:**

Dormancy of buds is a critical developmental process that allows perennial plants to survive extreme seasonal variations in climate. Dormancy transitions in underground crown buds of the model herbaceous perennial weed leafy spurge were investigated using a 23 K element cDNA microarray. These data represent the first large-scale transcriptome analysis of dormancy in underground buds of an herbaceous perennial species. Crown buds collected monthly from August through December, over a five year period, were used to monitor the changes in the transcriptome during dormancy transitions.

**Results:**

Nearly 1,000 genes were differentially-expressed through seasonal dormancy transitions. Expected patterns of gene expression were observed for previously characterized genes and physiological processes indicated that resolution in our analysis was sufficient for identifying shifts in global gene expression.

**Conclusion:**

Gene ontology of differentially-expressed genes suggests dormancy transitions require specific alterations in transport functions (including induction of a series of mitochondrial substrate carriers, and sugar transporters), ethylene, jasmonic acid, auxin, gibberellic acid, and abscisic acid responses, and responses to stress (primarily oxidative and cold/drought). Comparison to other dormancy microarray studies indicated that nearly half of the genes identified in our study were also differentially expressed in at least two other plant species during dormancy transitions. This comparison allowed us to identify a particular MADS-box transcription factor related to the *DORMANCY ASSOCIATED MADS-BOX *genes from peach and hypothesize that it may play a direct role in dormancy induction and maintenance through regulation of *FLOWERING LOCUS T*.

## Background

Leafy spurge (*Euphorbia esula *L.) is a perennial range land weed that infests the great plains of the US and Canada. This invasive and noxious weed maintains its perennial growth habit through the seasonal production of numerous underground adventitious buds on the lateral roots and crown (an underground portion of the stem derived originally from the hypocotyl). Crown and root buds of leafy spurge are capable of manifesting the three types of dormancy (para-, endo- and ecodormancy) described by Lang et al. [1]. In general, new leafy spurge crown buds become visible in late May to early June, following flowering, and progressively enlarge throughout the summer and fall seasons [2]. These crown buds, once formed, will not initiate new shoot growth unless the top of the plant is destroyed. Thus, initiation of new shoot growth is inhibited in newly formed crown and root buds during the growing season (usually from April to October) by mechanisms akin to apical dominance, or paradormancy. Studies on both crown and root buds indicate that two separate signals produced by the aerial portion of the plant are capable of maintaining bud dormancy [3]. These signals are auxin, likely acting through previously described mechanisms [4,5] and sugar acting through ABA/GA signalling that appear to regulate the G1 to S transition of the cell cycle [6,7].

Depending on yearly environmental conditions, crown buds of leafy spurge transition from paradormancy through endodormancy and into ecodormancy between early October and the middle of November [2]. This transition is important since early frosts could destroy the aerial portion of the plant, and thus initiate new shoot growth which would in turn be vulnerable to later frosts. Indeed, plants which are moved into growth-conducive conditions following the transition to endodormancy will not readily initiate new growth even if the above-ground portion of the plant is removed, suggesting that signals within the buds themselves inhibits growth. After an extended period of cold temperatures (usually coinciding with an average bare soil temp of 0°C which generally occurs by late November or early December in the location tested), crown buds are released from endodormancy and concomitantly become flowering competent [2]. However, by this time, consistently cold winter conditions induce a state of ecodormancy which blocks new shoot growth from the buds until spring conditions allow for renewed growth. We are interested in understanding the physiological processes involved in signals regulating the transitions between para-, endo- and ecodormancy.

There is emerging but incomplete information on the mechanisms by which dormancy transitions occur in adventitious buds of perennials. Chouard [8] noted commonalities between environmental signals and conditions regulating both vernalization (and hence flowering competence) and breaking of endodormancy. These observations have been recently reiterated and extended to develop a hypothesis that the signalling pathways regulating endodormancy induction and breaking might share components with those regulating flowering competency [9,10]. Indeed, work by Böhlenius [11] has demonstrated that constitutive expression of a flowering regulatory gene in poplar (*Populus trichocarpa*) *FLOWERING LOCUS T *(*FT*), is sufficient for preventing seasonal growth cessation induced by short day conditions. Likewise, over-expression of *PHYA *prevented short day-induced repression of *PtFT2 *in the leaves, and *CENTRORADIALIS-LIKE 1 *(*CENL1*), a gene related to *FT *but with opposite effect on flowering, in the rib meristem [12]. Indeed, the same study went on to show that *PHYA *over-expression prevented endodormancy *per se*. Combined, these data suggest that short day-induced growth cessation is the first step towards endodormancy induction, and that this first step can be impacted by expression of *FT *and related genes.

A mutation in peach caused by a deletion of a locus containing several tandomly duplicated MADS-box transcription factors, known as *evergrowing *(*evg*), prevents dormancy induction [13-15]. Similar genes were differentially expressed following dormancy transitions in raspberry (*Rubus idaeus *L.) and apricot (*Prunus mume*) [16,17]. These MADS-box genes (named *DORMANCY ASSOCIATED MADSBOX *or *DAM *genes) are related to *SHORT VEGETATIVE PHASE *(*SVP*) and *AGAMOUS LIKE24 *(*AGL24*) of *Arabidopsis *but form a separate clade within this group [15]. In *Arabidopsis*, mutations in *SVP *promote early flowering [18]. Interestingly, experimental evidence indicates that *SVP *negatively regulates expression of *FT *in *Arabidopsis *by binding to its promoter [19]. *AGL24*, a floral promoter, is up-regulated during vernalization [20]. Analysis of publicly available microarray data indicate that both *AGL24 *and *SVP *are preferentially expressed during short day (SD) conditions relative to long day in micro-dissected apical tissue harvested 0, 3, 5, and 7 days after the shift to LD. Thus, *SVP *and *AGL24 *are regulated by environmental conditions known to impact bud dormancy in perennial species.

In addition to *DAM *genes and *FT/CENL1 *with known or suspected involvement in bud dormancy/growth, several studies have been done on the impact of various dormancy transitions on the whole transcriptome of buds in kiwi fruit (*Actinidia deliciosa*), grape (*Vitis riparia*), raspberry, potato (*Solanum tuberosum*), and in the terminal buds and cambial meristem of poplar (*Populus tremuloidies*) [21,16-25], Marion Wood-personal communication. These studies have shown that dormancy signals impact numerous physiological processes including cell division, oxidative stress, and flavone biosynthesis. Additionally, these studies have demonstrated changes in expression patterns of numerous known regulatory genes that could play a vital role in dormancy transitions, or in regulating physiological processes impacted by dormancy transitions. However, no comparison of these bud dormancy data sets has yet been done.

Understanding the molecular mechanisms involved in regulating dormancy transitions in leafy spurge should provide information needed to control this invasive weed. Additionally, as there appears to be considerable conservation in the mechanisms regulating dormancy in other perennial systems, information gained on the mechanisms regulating dormancy transitions in leafy spurge could be used to develop means to control this critical process in other perennial crops, horticultural species, and weeds. We used newly developed high density cDNA microarrays which contained >19,000 unigenes sequences from leafy spurge. Additionally, the arrays contained >4,000 unigenes from cassava (*Manihot esculenta *Crantz), a related species, that generally hybridize to leafy spurge cDNAs [26] but which were not present in the leafy spurge EST collection. This array was used to follow transcriptome changes associated with dormancy transitions in leafy spurge over a 5 year period. Changes associated with each specific dormancy transition (para to endo and endo to eco) are noted, and commonalities between these results and other transcriptomic analyses of dormancy were identified.

## Results

### Transcriptome analysis identifies differentially expressed genes

**Figure 1:**
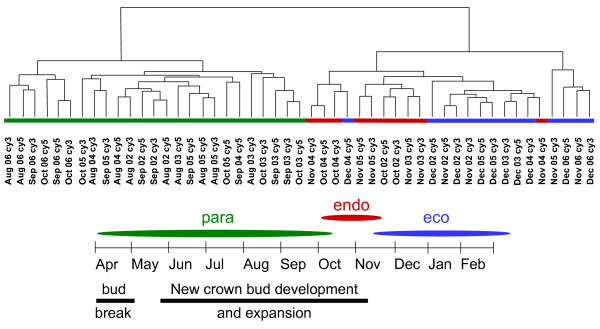
**Cluster analysis of expression data.** Green lines indicate samples designated as paradormant, red lines indicate samples designated as endodormant, and blue lines indicate samples designated as ecodormant. Monthly representation of bud development growth and dormancy transitions is shown below.

Changes in the transcriptome of leafy spurge crown buds samples, collected monthly from August to December for 5 consecutive years (2002–2006), were obtained by microarray analyses (GEO accession # GSE8849). From nearly 22,000 different unigene probes that showed consistent hybridization, about 1400 were identified as differentially expressed (p value < 0.005) based on ANOVA of samples grouped by month. Cluster analysis of these 1400 genes indicated that the monthly samples fell into two main groups (Figure 1). One group contained crown bud samples collected in August and September along with three crown bud samples collected in October. The remaining cluster was roughly divided into four sub-clusters; two containing nearly all of the December crown bud samples and several November samples, with the remaining clusters made up of a mix of October and November crown bud samples. The same clustering of samples based on expression was observed using principle component analysis and explained the majority of the variation observed in the data set (data not shown).

There is known yearly variability associated with the transition of crown buds into and out of endodormancy (Anderson J.V., personal communication). Thus, these results were interpreted to mean that the cluster containing all the August and September samples represented crown buds still in paradormancy. Likewise, the clusters containing December and November samples were considered to represent buds that were ecodormant and flowering competent. The remaining sub-clusters were considered to be endodormant. P-values and q-values based on differences between samples from the three dormancy states identified 999 genes with significant p-values (p < 0.005).

Probable *Arabidopsis *orthologues, MIPS designations, KOG#, GO annotations, and BLASTX hits were determined for these probes when possible (see Additional file 1). GO annotations indicated genes involved in jasmonic acid signalling, unidimensional cell growth, and transport were significantly over-represented among the differentially-expressed genes (Table 1). MIPS annotation suggested that genes involved in protein fate, protein synthesis, cellular communication, energy, protein binding, development, and cell fate were the most significantly over-represented (Table 2).

**Table 1:** Identification of over- and under-represented gene ontology (GO) terms in differentially-expressed gene set.

GO_Biological function	number of elements on the array	number of elements differentially expressed	number of elements expected to be differentially expressed	fold over or under represented	p-value	Adjusted p-value
response to jasmonic acid stimulus	46	12	1.83	6.63	1.45E-07	3.93E-06
unidimensional cell growth	15	7	0.60	11.86	6.96E-07	1.88E-05
Transport	317	32	12.61	2.57	1.19E-06	3.23E-05
signal transduction	91	9	3.62	2.51	0.01	0.26
response to oxidative stress	61	7	2.43	2.92	0.01	0.27
biological process unknown	5116	231	203.57	1.15	0.01	0.27
Metabolism	507	31	20.17	1.55	0.01	0.29
circadian rhythm	15	3	0.60	5.08	0.02	0.52
Growth	8	2	0.32	6.35	0.04	1
extracellular matrix organization and biogenesis	1	1	0.04	25.42	0.04	1
cell death	9	2	0.36	5.65	0.05	1
response to hormone stimulus	2	1	0.08	12.71	0.08	1
response to temperature stimulus	2	1	0.08	12.71	0.08	1
response to stress	43	4	1.71	2.36	0.09	1
response to cold	3	1	0.12	8.47	0.11	1
response to wounding	18	2	0.72	2.82	0.16	1
Aging	20	2	0.80	2.54	0.18	1
response to auxin stimulus	22	2	0.88	2.31	0.21	1
gibberellic acid mediated signaling	10	1	0.40	2.54	0.33	1
sugar mediated signaling	12	1	0.48	2.12	0.38	1
Biosynthesis	105	5	4.18	1.21	0.40	1
defense response	158	7	6.29	1.13	0.43	1
response to water deprivation	15	1	0.60	1.69	0.45	1
embryonic development (sensu Magnoliophyta)	23	1	0.92	1.11	0.60	1
response to heat	30	1	1.19	-1.18	0.70	1
photosynthesis	37	1	1.47	-1.46	0.77	1
Undetermined	18420	631	732.96	-1.15	1	1

The number of differentially-expressed genes with a given GO annotation present on the array was used to determine the expected number of differentially-expressed genes with that annotation. The number of observed differentially expressed genes was compared to the expected number to provide a fold over or under representation. P-values and false discovery rates (adjusted P-values) were identified.

**Table 2:** Analysis of over- and under-represented MIPS annotations terms for differentially-expressed gene set

MIPS FUNCTION	number of elements on the array	number of elements differentially expressed	number of elements expected to be differentially expressed	fold over or under represented	p-value	adjusted p-value
SUBCELLULAR LOCALIZATION	1263	3	50.25	-16.75	0	0
PROTEIN FATE (folding, modification, destination)	633	6	25.17	-4.20	9.11E-07	1.64E-05
PROTEIN SYNTHESIS	525	4	20.88	-5.22	1.18E-06	2.12E-05
CELLULAR COMMUNICATION/SIGNAL TRANSDUCTION MECHANISM	741	9	29.47	-3.27	2.26E-06	4.07E-05
ENERGY	439	3	17.48	-5.83	3.55E-06	6.38E-05
PROTEIN WITH BINDING FUNCTION OR COFACTOR REQUIREMENT (structural or catalytic)	618	7	24.58	-3.51	6.71E-06	1.21E-04
DEVELOPMENT (Systemic)	261	1	10.39	-10.39	2.67E-05	4.8E-04
CELL FATE	256	2	10.19	-5.09	3.79E-04	6.82E-04
INTERACTION WITH THE CELLULAR ENVIRONMENT	146	2	5.79	-2.90	0.02	0.37
BIOGENESIS OF CELLULAR COMPONENTS	542	12	21.58	-1.80	0.01	0.17
CELL CYCLE AND DNA PROCESSING	289	7	11.49	-1.64	0.06	1
TRANSCRIPTION	726	24	28.87	-1.20	0.17	1
CELL RESCUE, DEFENSE AND VIRULENCE	577	21	22.977	-1.09	0.33	1
CELLULAR TRANSPORT, TRANSPORT FACILITATION AND TRANSPORT ROUTES	758	28	30.17	-1.08	0.34	1
CLASSIFICATION NOT YET CLEAR-CUT	1072	44	42.66	1.03	0.59	1
UNCLASSIFIED PROTEINS	14494	711	565.43	1.26	1	1
METABOLISM	1737	111	69.13	1.61	1	1
STORAGE PROTEIN	30	4	1.20	3.34	0.97	1

The number of differentially-expressed genes with a given MIPS annotation present on the array was used to determine the expected number of differentially-expressed genes with that annotation. The number of observed differentially expressed genes was compared to the expected number to provide a fold over or under representation. P-values and false discovery rates (adjusted P-values) were identified.

MAPMAN analysis indicated that genes involved in metabolic pathways for glycolysis, transport (calcium and major intrinsic proteins), protein synthesis initiation, auxin responses, and redox regulation were the most over-represented (Table 3). Surprisingly, although glycolysis genes were both over-represented and generally induced through the dormancy transitions, almost none of the genes involved in the TCA cycle were present among the differentially-expressed genes.

**Table 3:** MAPMAN analysis of over- and under-represented physiological processes for differentially-expressed gene set.

BIN	Function	elements	p-value
4	glycolysis	8	0.0001
34.19.1	transport major intrinsic proteins, PIP	4	0.0090
21	redox, regulation	6	0.0119
29.2.3	protein synthesis, initiation	4	0.0308
34.21	transport, calcium	3	0.0451
17.2	hormone metabolism, auxin	3	0.0464

The probability (p-value) that the number of genes involved in any given function (elements) were present by chance was obtained using the default parameters of the MAPMAN program.

BLASTX searches for possible gene function indicated that numerous genes with similarity to known or suspected cold stress response genes were up-regulated in fall and winter. Likewise, numerous genes with similarity to known or suspected cell division genes were generally down-regulated through seasonal transitions from summer to winter. There were also many genes with similarities to transport associated functions that were preferentially expressed as dormancy progressed from the fall through the winter (See Additional file 2). Some specific differentially-expressed genes of note included a *SWITCHING2/SUCROSE NON_FERMENTING2 *(*SWI2/SNF2*) which encodes a protein known to play a role in chromatin remodelling [27], were steadily down-regulated during seasonal dormancy progression. Genes similar to *EARLY FLOWERING 4 *(*ELF4*), *GIGANTEA *(*GI*), *CONSTANS *(*CO*), *FLAVIN BINDING KELCH REPEAT F-BOX1 *(*FKF1*), *CIRCADIAN CLOCK ASSOCIATED1 *(*CCA1*), *PHYA*, and several circadian-regulated *PSEUDO-RESPONSE REGULATORS *(*PRR*s) that are possible components of the circadian clock regulatory pathway [28,29] are all up-regulated during the seasonal progression (from paradormancy through to ecodormancy).

**Figure 2:**
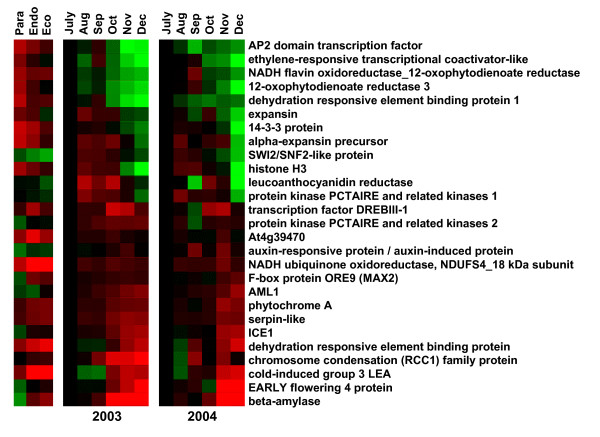
**Confirmation of microarray expression profile by RT-PCR.** RT-PCR analysis using crown bud RNA collected monthly during 2003 and 2004 was done on 27 selected genes. Relative gene expression levels are shown with high expression represented by red and low expression represented by green.

Expression of a subset of differentially-expressed genes was confirmed by quantitative RT-PCR (Figure 2). Comparison of these genes suggests that the general trends in expression observed by microarray analysis are similar but somewhat muted as compared to that observed by RT-PCR. 100% of the 27 genes tested by RT-PCR showed similar trends to those observed using microarray analysis, indicating the robustness of our analysis.

### Gene expression characteristics of different dormancy states

Analyses were done to identify genes that showed differential expression unique to specific dormant states (See Additional file 3a–c). Only 16 genes were preferentially expressed during endodormancy (see Additional file 3b). One of these contains a helix-loop helix motif characteristic of DNA binding transcription factors, and another was similar to DROUGHT RESPONSIVE ELEMENT BINDING III (DREBIII), a transcription factor known to play a role in drought and cold responses in plants. Only 5 genes were significantly down-regulated specifically during endodormancy. Genes involved in flavanoid biosynthesis were relatively highly expressed during paradormancy and then down-regulated during endo- and ecodormancy. Likewise, several growth regulating genes such as *GROWTH REGULATING FACTOR 5 *(*GRF5*), a *RETINOBLASTOMA*-like, and *ARABIDOPSIS MEI2 LIKE1 *(*AML1*) are also preferentially expressed in ecodormant buds (see Additional file 2).

### Comparison to other dormancy microarray studies identifies common signals and processes

There have been four other published microarray studies on bud dormancy in raspberry [16], poplar [23], potato [25] and grape [21]. Additionally, there are as yet unpublished studies completed on kiwifruit (Marion Wood et al., personal communication), and studies on seasonal dormancy in poplar cambial meristems [22,24]. TBlastX comparisons between the genes represented on the arrays from these various datasets (see Additional file 4) indicated that 431 leafy spurge genes were differentially expressed in both poplar buds induced into dormancy by SD and in our leafy spurge dataset (240 showed the same expression trend in the transition from paradormancy into endodormancy). Likewise, 722 were differentially expressed in both potato tuber meristems during the transition from endodormancy directly to growth and during the endodormancy to ecodormancy transition in our leafy spurge dataset. In addition, 460 were differentially expressed in at least three species, and 11 were differentially expressed in all 5 species.

The putative function of the 110 differentially-expressed genes in four or more species suggest cell cycle, transport, stress, and GA responses are important conserved processes affected by dormancy transitions. An analysis of genes differentially expressed in three or more species also identified these same processes and additional common features included circadian-regulated genes, ABA/auxin/light regulatory and responsive genes, and genes encoding transport functions and flavanoid biosynthesis. Differentially regulated transcription factors found in 3 or more species included the *DAM *genes, *AGAMOUS LIKE63*, *BLIND*, *DREB/CBF*, *INDUCER OF CBF EXPRESSION *(*ICE1*), *HOMEOBOX3 *(*HOX3*), 4 *MYB*, 3 *MYC*, *HEXAMER BINDING PROTEIN-1b *(*HBP-1b*), *HOX 4*, *WRKY *(A1244, 30, 53), and nine different zinc finger-encoding genes. One MYB and one zinc finger-containing transcription factor were among those genes noted as commonly differentially-expressed in poplar buds, cambial meristems, and seed dormancy transitions [23]. Only 41 other genes were differentially expressed during dormancy transitions in both *Arabidopsis *seed and leafy spurge buds, and of these, only 16 were expressed in a similar manner in response to dormancy transitions (data not shown). No obvious signals or pathways were common to both seed and bud dormancy transitions were detected.

### ABA levels drop following endo- to ecodormancy transition

ABA levels are correlated with bud dormancy in several species and tend to drop as buds transition through ecodormancy [30-32]. However, very few putative ABA-regulated genes were down-regulated (see Additional file 2). To determine if ABA levels changed between endo and ecodormancy, ABA levels were measured over two years in Oct. and Dec. crown bud samples. The results show a significant decrease in ABA between Oct. and Dec., in line with other observations (Table 4).

**Table 4:** Average ABA levels (ng/gm f-wt) in buds from Oct. and Dec. 2005 and 2006.

Oct. 2005	Oct. 2006	Dec. 2005	Dec. 2006	P value
97.08	94.4	14.04	34.65	0.008

P value equals probability that Oct. and Dec. samples are equivalent based on T-test from two to three independent samples from each time point.

### DAM and FT-like are reciprocally and differentially expressed during transition from para-through ecodormancy in leafy spurge

**Figure 3:**
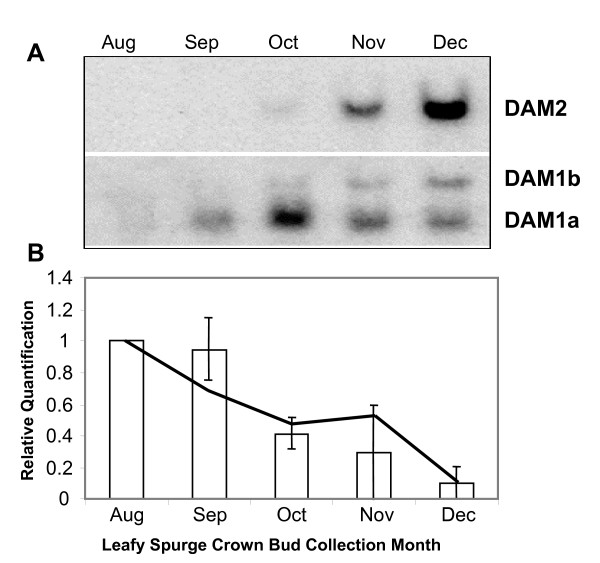
**Comparison of DAM gene and FT expression through dormancy transitions.** A) northern blot analysis of *DAM1 *and *DAM2 *expression over a representative seasonal time course (2004) in leafy spurge. B) Bars show relative RT-PCR analysis of *FT *expression over 3 years with the line showing expression during the 2004 year. Error bars represent standard deviation of yearly expression pattern.

There is growing evidence that *DAM *genes play a role in regulating dormancy transitions. Two clones (DV112957 and DV114890) representing different *DAM *genes were present on our microarrays and both were found to be differentially regulated (see Additional file 2). Probes specific to the 3' end of these genes were developed and used to probe northern blots to confirm the differential expression observed from the microarray results. The leafy spurge *DAM1a *gene was strongly up-regulated only in endodormant buds whereas *DAM2 *was induced during endodormancy and maintained at high levels through ecodormancy (Figure 3). Because the *DAM1 *probe was shown to have minimal but detectable cross-hybridization to a *DAM2 *clone (data not shown), it is unclear if the upper band (*DAM1b*) from the blot probed with *DAM1 *is an alternate splice product of *DAM1 *(of which several are suspected) or if it is the result of cross-hybridization to other *DAM *family members.

*FT *over-expression prevents seasonal growth cessation required for endodormancy induction in poplar [11]. Because *DAM *genes are very similar to *SVP*, and *SVP *negatively regulates *FT *in *Arabidopsis *[19], we hypothesize that the *DAM *genes may negatively regulate *FT *expression. As an initial test of this hypothesis, we characterized and compared the expression of an *FT*-like gene from leafy spurge to that of the *DAM *genes to determine if they were reciprocally expressed. Quantitative RT-PCR on the same RNA used for northern blot expression analysis of *DAM1 *and *DAM2 *demonstrated that the *FT*-like gene was down-regulated in crown buds of leafy spurge concomitantly with the induction of *DAM *gene expression (Figure 3).

## Discussion

### Cell cycle and cold responsive genes are differentially expressed during dormancy transitions

**Figure 4:**
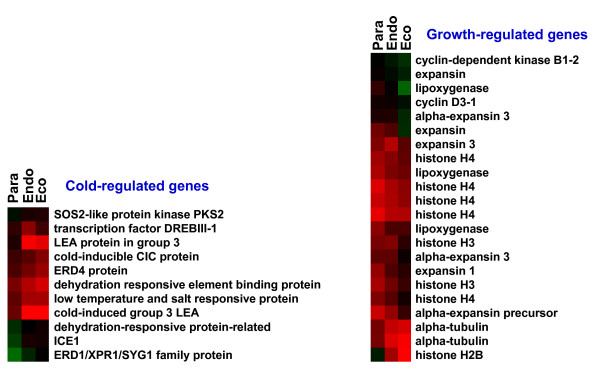
**Heat map diagram of relative gene expression levels for probable cold-regulated and growth-regulated genes. **Red color indicates relative increase in expression, and green color represents relative decrease in expression.

Approximately 50% of the differentially-expressed genes identified by our microarray analysis show a consistent pattern of incremental induction or repression during the transitions from paradormancy through to ecodormancy. However, about a third of the genes showed their greatest change in expression in the transition from the paradormant state to the endodormant state (see Additional file 3a). The timing of this transition usually coincides with decreasing night time temperatures. Thus, it was not surprising to see a majority of the genes involved in cold hardening such as *ICE1*, *DREB *and the type 3 *LEA *genes are up-regulated during the transition from para- to endodormancy (Figure 4, see Additional file 3a). Likewise, many of the genes involved in cell division are down-regulated during the transition between endodormancy and ecodormancy (see Additional file 3c). Previous studies have shown that *HISTONE H3 *is strongly down-regulated late in endodormancy or early in ecodormancy [2]. The microarray results show a similar pattern of expression for most of the growth-responsive genes (Figure 4). Combined, these expected results suggest that the analysis of the arrays was appropriate for identifying differentially-expressed genes.

Intriguingly, Espinosa-Ruiz [33] found that endodormancy induction brought about reduced transcript levels of cell cycle genes, but under conditions of ecodormancy these same genes were not repressed. However, unlike our study, ecodormancy in the Espinosa-Ruiz study was brought about by SD, whereas cold was the primary signal maintaining ecodormancy in our study. Thus, the different environmental conditions that maintain ecodormancy in these two studies appear to have different impact on cell division. However, data collected by Druart [24] on cambial meristems indicated that cell cycle genes were down-regulated during the transition from growth to endodormancy, and that the levels then stayed low or increased slightly during ecodormancy.

Several notable growth regulating genes that were preferentially expressed in ecodormant buds (see Additional file 2) included a *RETINOBLASTOMA*-like (*RB*-like) protein, *GROWTH REGULATING FACTOR5 (GRF5*), and *ARABIDOPSIS MEI2-LIKE1 *(*AML1*). *RB *is involved in cell cycle inhibition and acts both through sequestration of several *E2F *transcription factor family members-positive regulators of cell division, and through chromatin modification of key cell cycle regulatory genes [34,35]. *GRF5 *encodes a putative transcription factor that is involved in regulating leafy growth and morphology [36] and *AML1 *is a *MEIOSIS2*-like (*MEI2*-like) RNA-binding protein gene, required for meiosis and vegetative development and whose protein interacts with *RAPAMYCIN-TOR *(*RAPTOR*), another protein involved in regulating cell division [37,38]. It is surprising that *RB*-like, *GRF5 *and *AML1 *are preferentially expressed in a dormancy state where many of the genes involved in active growth such as histones, cyclins and cyclin dependent kinases are repressed. Our observations can be interpreted to suggest that these particular growth-regulators are activated by conditions besides growth induction, and may be turned on primarily in response to signals regulating growth competency such as extended cold. Extended cold is known to induce growth competency in many perennial buds through unknown mechanisms. These genes may provide a convenient place to begin investigating this process. It will also be of interest to identify some of the targets regulated by these genes and determine if they are also preferentially expressed in dormant buds, or if they are maintained in a suppressed state.

### Circadian responses associated with seasonal dormancy transitions

The observation that several circadian regulatory genes are differentially expressed during dormancy transitions was not surprising (see Additional file 2). However, the fact that many of them were differentially expressed in underground buds and that all of them were up-regulated following the paradormancy to endodormancy transition was initially surprising. However these results are consistent with studies in chest nut (*Castanea sativa *Mill.) that indicated constitutive expression of circadian clock genes during winter dormancy [39]. Although crown buds do appear to turn green and activate several photosynthetic genes such as *LIGHT HARVESTING CHLOROPHYLL A/B-BINDING PROTEIN *during ecodormancy [2,40], it was surprising to see changes in putative circadian responses in organs that are often below the soil surface. Also, the coordinated expression changes in genes such as *GIGANTEA *(*GI*), *FLAVIN BINDING KELCH REPEAT F-BOX1 *(*FKF1*), *CONSTANS *(*CO*), and *EARLY FLOWERING4 *(*EFL4*) would be expected, as would expression of *PHYA*, *CCA1*, and the *PRR*s [29]. However, *GI*, *FKF1*, and *EFL4 *are all directly or indirectly negatively-regulated by *CIRCADIAN CLOCK ASSOCIATED1 *(*CCA*) [29]. More work is needed to determine if these genes are functional orthologues of the *Arabidopsis *genes and to determine if other environmental, physiological, or developmental signals are modifying the expected expression pattern of these genes in the underground buds.

### Conserved regulation of flowering and dormancy genes in perennials

Perhaps the most interesting result from these studies is the finding that *DAM *genes are differentially expressed during dormancy transitions in leafy spurge, poplar, potato, and raspberry. The importance of this differential expression was not obvious when each study was considered on its own. Initial comparisons between our data set and the data set from raspberry identified similar *MADS*-box genes as differentially expressed in both species. Thus, an effort was taken to catalogue *DAM *gene expression in other species as studies were made available. In raspberry, a *DAM*-like gene was down-regulated upon dormancy release [16]. Another *DAM*-like gene was reported to be up-regulated in poplar after 4 weeks under dormancy-inducing SD conditions (albeit not significantly) and was significantly up- or down-regulated in *ABI3 *under- and over-expressing lines respectively [23]. Moreover, the same poplar gene was significantly up-regulated in the cambial meristem upon endodormancy induction and then down-regulated upon the transition from endodormancy to ecodormancy [24]. A close relative, *MADS16*, was identified as up-regulated in potato tuber meristems during the transition from endodormancy to growth [25]. However, our results provide the first evidence that different members of this gene family may have variable expression patterns through seasonal dormancy transitions (Figure 3a).

Although conserved regulatory gene expression patterns are suggestive of a functional role, such data is only correlative. Direct evidence that *DAM *genes are regulating dormancy comes from work on the *evg *mutation in peach. The *evg *mutation prevents terminal peach buds from going dormant in the fall [13]. The locus containing the *evg *mutation has recently been cloned and sequenced [14,15]. Sequencing through this locus indicated that the main lesion was a deletion of a series of tandomly repeated copies of *DAM *genes. Our data highlights the fact that not only are these genes required for endodormancy induction, but that *DAM *genes are induced by environmental signals known to regulate endodormancy.

SD and cold temperatures regulate dormancy in a species specific manner. SD rather than cold temperature is the primary signal regulating endodormancy induction in poplar [41]. In some rosaceous species such as apple and pear, cold temperatures are the primary environmental signal inducing endodormancy [42]. Experiments are currently underway to determine the environmental parameters required for endodormancy induction in leafy spurge, however preliminary experiments suggest SD is not sufficient for endodormancy induction. However, it should be noted that there is evidence of synergistic cross-talk between SD and low-temperature signalling pathways [43], and thus it is possible, if not likely, that cold and day length signalling mechanisms regulating dormancy may be shared across species. It will be interesting to compare *DAM *gene regulation in species that primarily respond to either light or temperature signals for dormancy induction.

We hypothesize that DAM proteins regulate dormancy transitions through interaction with *FT*-like genes. In *Arabidopsis*, *SVP *may be involved in sensing changes in ambient temperatures and transducing this information to regulate flowering by inhibiting *FT *expression through binding of CArG motifs [19]. In poplar, over expression of *FT *inhibits growth cessation and bud set associated with seasonal bud dormancy [11]. Expression of *FT *or *FT*-like genes such as *CENL1 *are down-regulated in grape buds (Fennell, personal communication), poplar [12,23], and spruce [44] during induction of endodormancy. Our paradigm predicts that *FT *should be reciprocally expressed with *DAM *genes. However, no *FT *genes were present in the leafy spurge or cassava EST databases and thus not present on the microarrays.

As an initial test of our hypothesis, we cloned and characterized the expression of an *FT*-like gene from leafy spurge (Accession # EU707393). Several cDNA clones and a genomic clone (Accession # EU707394) contained the 3' end of *FT*-like genes (49% of the coding region). We suggest our clones represent *FT *orthologues rather than orthologues of the closely related gene *TFL *because 1) the *FT*-like genomic clone contained an intron with similar size and location to the last intron in the *Arabidopsis FT *gene, 2) a BLASTX search of the *Arabidopsis *database demonstrated that *FT *was the top blast hit (with a score of 2e-39) to our clones, 3) cluster analysis of DNA sequence placed our *FT*-like genes with other *FT *genes and away from related *TFL *genes, including several full-length *TFL*-like genes from leafy spurge (Accession #s DV157402, DV135948, and DV121805), and 4) our *FT*-like gene is expressed preferentially in leaves and is diurnally regulated as would be expected of a functional *FT *orthologue (see Additional file 5, 6, 7). Work is continuing to clone full length copies of *FT*-like genes from leafy spurge and confirm their functionality. However, consistent with our hypothesis, the *FT*-like gene is reciprocally regulated with expression of our *DAM *genes.

Some questions remain concerning the role of *FT *and *DAM *genes through dormancy transitions. Although it seems likely that *DAM *genes and *FT *may play a direct role in seasonal growth cessation and possibly in the transition from paradormancy to endodormancy, it should be noted that at least *DAM2 *is expressed well into ecodormancy. Likewise, *FT*-like is also down-regulated well into ecodormancy. Thus, the *DAM *gene and *FT*-like probably do not play any role in the transition to growth competence that occurs during the shift from endodormancy to ecodormancy. This suggests that there are other signals and mechanisms involved in controlling the transition from endo- to ecodormancy and concurrent induction of floral and growth competence in leafy spurge.

Work is currently underway to identify the *cis*- and *trans*-acting signals regulating *DAM *gene expression in leafy spurge and to study the impact of constitutive and dominant negative regulation of the leafy spurge *DAM *genes on bud dormancy and flowering in leafy spurge and several heterologous systems. Likewise, work is underway to functionally characterize and to clone the regulatory regions of the *FT*-like genes from leafy spurge and to determine if they contain the conserved CArG motifs, and to determine if these *FT*-like genes are directly regulated by *DAM *genes from leafy spurge during induction of dormancy.

### Chromatin remodelling in dormancy transitions

Given the similarities between the environmental and molecular signals regulating both flowering and dormancy, it is tempting to speculate that the same mechanisms regulating chromatin remodelling processes and genes regulating vernalization and flowering competence in *Arabidopsis*, also regulate the re-initiation of growth competence in vegetative buds of perennials following extended cold treatments. Numerous genes involved in chromatin remodelling are differentially expressed during dormancy transitions in leafy spurge and other species. A member of the chromosome condensation (RCC1) family of proteins which interact with histones is strongly up-regulated in leafy spurge crown buds during induction of endodormancy. Interestingly, a similar protein is down-regulated in potato buds transitioning from endodormancy to growth [25]. Additionally, RB-related proteins, also affecting chromatin remodelling as previously discussed, are differentially-expressed in leafy spurge, poplar and potato (see Additional file 8).

Ruttink [23], found four different chromatin remodelling genes that were significantly up-regulated prior to endodormancy induction, but considered the induction to be too early to impact the transition to endodormancy. However, in their studies, they did not look at dormancy release, and thus it is impossible to determine if these or other chromatin remodelling genes might be differentially expressed in response to extended cold treatments. Law and Suttle [45], noted that dormancy release in potato was concomitant with increased histone acetylation. Likewise, chromatin remodelling was suggested by Druart [24] to explain the large scale changes in transcription observed in poplar cambial meristems during seasonal dormancy transitions. In both leafy spurge and poplar, a *SWI2/SNF2*-like gene similar to At5g66750, was significantly down-regulated (steadily through dormancy transitions in leafy spurge and after 4 weeks of SD in poplar). SWI2/SNF2 proteins are needed as part of a complex known to modify chromatin and enhance expression of specific genes [46].

### Auxin responses in seasonal dormancy transitions

Other comparisons between the transition from paradormancy to endodormancy and from endodormancy to ecodormancy indicate that five of the six genes with blast hits to genes involved in auxin responses show the greatest differential expression during the paradormancy to endodormancy transition. MAPMAN analysis also indicated an increase in expression of genes involved in auxin metabolism (Table 3). This also might be expected since as the plants senesce during the transition into endodormancy, auxin transport from the above ground tissue would be reduced. Indeed, a similar argument was made to explain observed induction of a *DORMANCY-ASSOCIATED, AUXIN-REPRESSED *(*DAAR*) gene in crown buds of leafy spurge [2]. *DAAR *genes are known to be repressed by auxin in several plant systems [47,48]. These observations are consistent with findings of Schrader [22], suggesting that auxin responses may be modulated in both cambial meristem and adventitious buds during endodormancy, and Li [49] that indicated auxin levels were reduced in terminals buds of silver birch (*Betula pendula*) during short-day-induced dormancy.

BLASTX analysis of the differentially-expressed genes indicated down-regulation of genes involved in flavanoid biosynthesis in buds that have passed from paradormancy through to ecodormancy (see Additional file 2). Flavanoids have been implicated in inhibition of polar auxin transport [50-53]. The ability of buds to export auxin has been hypothesized to be the driving force behind the signals regulating apical dominance [54]. In potato buds, auxin levels steadily increase during dormancy and then drop off after dormancy is released [55]. Combined, these data suggest that as the buds lose apical dominance, they may be preparing to export auxin even when the buds are not transitioning into an actively growing state. These data support earlier microarray studies on root buds of leafy spurge which indicated that flavanoid biosynthetic genes were down-regulated following release from paradormancy [56].

It should be noted, however, that although several *AUX/IAA*-like genes are down-regulated during the transition from paradormancy to endodormancy, a gene with similarity to *MORE AXILLARY BRANCHES *(*MAX2*) that encodes a protein involved in inhibition of auxin transport [57,54], is up-regulated (see Additional file 2). Thus, it appears that there are opposing mechanisms involved in auxin production, transport, and perception acting simultaneously during dormancy transitions, and more work will be needed to ascertain the relevance of this hormone in seasonal dormancy transitions.

### JA, ethylene, and ABA responses in seasonal dormancy transitions

Many of the genes associated with cold, drought and ABA responses are preferentially expressed later during endo- and ecodormancy (Figure 4). From work in poplar and potato, there appears to be an emerging picture that a transient spike in ethylene (or ethylene perception) precedes, and is necessary for, the initiation of endodormancy [58,23]. A role of ethylene in ABA-induced growth inhibition in systems other than bud endodormancy has been previously hypothesized [59]. Work on ABA accumulation in *Citrus *suggests that ethylene may directly induce the key ABA biosynthetic gene *9-CIS-EPOXYCAROTENOID DIOXYGENASE *(*NCED1*) [60]. Our data appears consistent with the above theory in that there are at least 10 genes associated with ethylene production or ethylene responses that are most highly expressed during paradormancy, but which were repressed later during endo- and ecodormancy.

Intriguingly, ABA levels were elevated during endodormancy and dropped following the transition to ecodormancy (Table 4). Loss of ABA through ecodormancy has been observed in several plant species [30-32]. However, all but four expected ABA-regulated genes are induced during the endo- to ecodormancy transition (see Additional file 2), and of the four that showed reduced expression, only one (DREBIII) is significantly down-regulated. A possible explanation for this observation is that many ABA-regulated genes are also cold-regulated, and there is evidence that cold and ABA regulates these genes through independent pathways [61].

The high numbers of JA-regulated genes suggest a possible role for this hormone in seasonal dormancy progression (see Additional file 2). JA is generally associated with wounding, and there are also reports that JA acts synergistically with ethylene [62-64]. JA is also known to induce vegetative storage protein expression in numerous plant systems [65-68]. Likewise, JA is known to induce tuber formation in potato [69]. Thus, increased JA perception during paradormancy might be needed to prepare the plants for winter. Vegetative storage proteins were also the most over-represented among the differentially-expressed genes based on MIPS FunCat designation (Table 2). A similar argument was noted for the increase in differentially-expressed JA-responsive genes during the transition into endodormancy in poplar cambial meristems [24].

## Conclusion

These results provide the first indication of changes in gene expression in underground buds of a herbaceous perennial associated with seasonal dormancy transitions. Changes in gene expression suggest numerous hormonal, physiological, and developmental responses occur during transitions from paradormancy through endodormancy and into ecodormancy including alterations in responses to ABA, JA, and auxin, and in cell cycle, cold-hardening, transport, and glycolysis. Additionally, comparison between the transcriptome changes observed in our experiments and those from similar dormancy transitions in other plant species allowed the identification of *DAM *genes that encode a specific group of MADS-box transcription factors associated with endodormancy induction. The fact that *DAM *genes are similar to another MADS-box gene (*SVP*) that negatively regulates *FT *in *Arabidopsis*, and the finding that over-expression of *FT *in poplar prevents seasonal growth cessation led to the hypothesis that induction of *DAM *genes may be required for inhibition of *FT *expression. In support of this hypothesis, *FT *expression in leafy spurge crown buds was co-ordinately down regulated as *DAM *gene expression was induced.

## Methods

### Plant material

A population of leafy spurge plants was initiated from cuttings of the same ND001 line used in several previous studies on leafy spurge bud growth and development [2]. Plant were maintained in an outside garden plot in Fargo, ND and supplemental water and fertilizer was added as needed for several years prior to initial bud collections. Crown buds were collected monthly (August through December from 2002–2006). Buds were always collected between 10:00 AM and 1:00 PM for all samples. Environmental data at and around the time of bud collections are noted (see Additional file 9). Bud samples were stored immediately in liquid N_2 _and then at -80 C until RNA extraction.

### ABA extraction and analysis

Each sample consisted of 0.5 to 0.6 gm of pooled buds collected in Oct. or Dec. 2005 and 2006. Samples were immediately frozen in liquid nitrogen. Two or more samples were analyzed in duplicate for each time point. The tissues were thawed at 4 C in 80% (v/v) aqueous acetone, homogenized, extracted, purified, and the ABA content quantified by HPLC-MS using an internal standard of 50 ng [^2^H]_6_-(+)-ABA (OlChemIm Ltd, Czech Republic) as described by Destefano-Beltran [70].

### RNA extraction and microarray analysis

Crown buds were ground to a fine powder in liquid N_2 _and RNA was extracted using the pine tree extraction protocol [71]. RNA quality and quantity was confirmed by spectrophotometry and denaturing agarose gels. Labelled cDNA was prepared from 30 μg of total RNA using the Alexa Fluor cDNA labelling kit (Invitrogen, Carlsbad, CA) according to manufacture's protocols. Labelled cDNAs were hybridized to a custom made 23 K element microarray that contained 19,808 unigenes from the leafy spurge EST database [72] and an additional 4,129 unigenes from a cassava EST database [73]. The cassava unigenes did not have any obvious similarity to available leafy spurge ESTs, however, many of them showed strong hybridization with labelled leafy spurge cDNA [26]. A rolling circle dye swap hybridization scheme [74] was used to compare gene expression between samples. Microarray hybridization was visualized using an AFFYmetrix 428 scanner (see Additional file 10) and spot intensities and background was quantified using Affymetrix Jaguar software. GeneMaths XT 5.1 software (Applied Maths Inc. Austin, TX) was used for statistical analysis and clustering of the dataset.

Hybridization intensities were log2 transformed, and arrays were centred and normalized against each other. Arrays were grouped by month and ANOVA was used to identify genes that were differentially expressed (p < 0.005). Cluster analysis of expression pattern for differentially-expressed genes was done to identify likely paradormant, endodormant and ecodormant samples. The entire data set was then regrouped according to dormancy state, and subjected to ANOVA again to identify genes that are differentially expressed (p < 0.005) through the dormancy transitions. To identify genes with significant differential expression during specific dormancy states, T-tests were used to determine p-values that a given gene was differential in one dormant state, but not differential between the other dormant states (ie. paradormant specific genes would have p < 0.005 between paradormancy and endodormancy and p > 0.005 between endodormancy and ecodormancy).

Putative *Arabidopsis *orthologues were identified by BLASTX homology with a cut off of 1.0 E-5. Likewise, TBLASTX was used to identify similar genes present on other microarrays used for dormancy studies (cut off of 1.0 E-5) (see Additional file 8). Where possible contigs represented by spotted cDNAs were used for the BLAST comparisons. AGI designations for *Arabidopsis *genes were used in conjunction with MAPMAN to identify biochemical and signalling pathways represented by the differentially-expressed genes.

### RT-PCR and northern blot expression analysis

Primers were designed (see Additional file 11) to specifically amplify genes identified as differentially expressed using sequences from the EST database[72,73]. cDNA from bud samples collected during the designated years was prepared, quantified, and checked for quality by separation on agarose gels. Equal amounts of cDNA were used in each reaction and reactions were run in triplicate. SYBR green and the endogenous ROX reference dye were used to determine relative CT values. For RT-PCR of the *FT*-like gene, the primer-probe combination was designed by Applied Biosystems Custom Taqman(R) Gene Expression Assay Service based on sequence from the genomic clone: reverse primer GCTGGTCTTGGACTCTCATACC, forward primer GGTGACTGATATTCCAGCAACTACT, and probe TCTCTTGCCCATAGCTTG. The probe was designed to span the last intron in the gene to eliminate signal from contaminating genomic DNA or un-spliced RNAs.

Probes for northern analysis were produced by PCR from full length cDNA clones of DAM1 (EU334633) and DAM2 (EU339320). Primers for *DAM1 *specific probes were (5') GAGTTATCTACTCTTTGTGATG and (3') CAATTGTCAACTATTTATTGGATGG and amplified a 260 bp fragment containing a portion of the coding region (of which 83 nt were common with *DAM2*) plus the 3' UTR. *DAM2 *primers were (5') TGACTCGGGTGATCGAAAG and (3') AGTCGCTCGTTCTCTTCC and amplified a 313 bp product covering the 3' portion of the coding region (none of which was homologous to *DAM1*). These fragments were radio-labelled and used to probe a northern blot containing equal loadings (10 μg each) of total crown bud RNA (collected monthly in 2004) separated on a denaturing agarose gel.

Accession numbers for sequence and gene expression data. GEO series # GSE8849, Genbank #s DAM1 – EU334633, DAM2 – EU339320, EeFT2L – EU707393, EeFT2gs – EU707394, EeFT10 – EU707395, EeTFL1-3 – DV157402, DV135948, and DV121805

## Authors' contributions

DPH conceived of the study, participated in its design, drafted the manuscript, and performed the microarray hybridization and analysis. WSC performed and analyzed the Real-time PCR data. JCS performed and analyzed the ABA quantification. JT assisted with the bioinformatics. JVA provided the plant material for these experiments and assisted in the experimental design.

## Supplementary Material

Additional file 1 **Differentially expressed genes.** List of differentially-expressed genes, gene expression data, and gene annotation. Click here for file

Additional file 2 **Differentially expressed genes specifically mentioned in text.** List of genes with functional annotations specifically mentioned in the text. Gene expression data, and gene annotation is noted for each. Click here for file

Additional file 3 **Genes differentially expressed specifically during para-endo- and ecodormancy.** List of gene expression data and gene annotation for genes with significant expression patterns during paradormancy (3a) endodormancy (3b) and ecodormancy (3c). Click here for file

Additional file 4 **Common differentially expressed genes from dormancy transitions in other species.** List of differentially-expressed genes with blastX hits to differentially-expressed genes in datasets from dormancy transitions for other plant species. Click here for file

Additional file 5 **Cluster analysis of FT and FT-like genes from various species.** Phylogenetic analysis of FT-like and TFL-like genes from leafy spurge and various other species. Nucleotide sequence data from four different *FT*-like genes obtained from leafy spurge (3'Race-EeFT10 (Accession # EU707395), amplification of genomic DNA-EeFTgs (Accession # EU707394, and amplification from cDNA-EeFT2lb (Accession # EU707393) were aligned to several FT-like and TFL-like genes from leafy spurge, poplar, castor bean, *Arabidopsis*, potato, tomato, and apple using ClustalX. Bootstrap values were generated as shown (1000 iterations). Click here for file

Additional file 6 **Sequence data from leafy spurge and *Arabidopsis *FT genes. **Genomic sequence from amplified region of FT-like gene from leafy spurge (top) and equivalent region from genomic DNA sequence of arabidopsis FT gene (bottom). Intron sequence is shown in bold lower case. Identical bases are noted in blue. Click here for file

Additional file 7 **Northern analysis of FT expression in leafy spurge.Supplemental Figure S3**: Northern analysis of RNA collected from leaf tissue approximately 7 hr after dawn (D) and 3 hr after dusk (N) from outdoor-grown flowering competent (F) and greenhouse-grown flowering incompetent (NF) plants. Northern blot was probed with P32 labelled amplified *FT*-like cDNA fragment (EeFTgb). Click here for file

Additional file 8 **All differentially expressed genes from dormancy transitions in other species.** List of all differentially-expressed genes in poplar, leafy spurge, potato, raspberry, and grape. Rows contain differentially expressed genes from represented species. All genes in a given row are matched with BLASTX scores of <10E-5. Click here for file

Additional file 9 **Collection dates and conditions. **Dates and weather conditions at and prior to the time of bud collections. Click here for file

Additional file 10 **Normalized expression data.** List of all spots on the microarray along with gene expression data and gene annotation. Click here for file

Additional file 11 **Primers and reaction conditions for quantitative RT-PCR.** List of primer sequences and PCR conditions used for RT-PCR confirmation of differential gene expression for selected genes. Click here for file
