# Supplementary figures and images for: Transcriptome analysis identifies novel responses and potential regulatory genes involved in seasonal dormancy transitions of leafy spurge (Euphorbia esula L.)
Source: BMC Genomics. 2008 Nov 12;9:536. doi: 10.1186/1471-2164-9-536 (PMC2605480; doi:10.1186/1471-2164-9-536)

## Slide 1
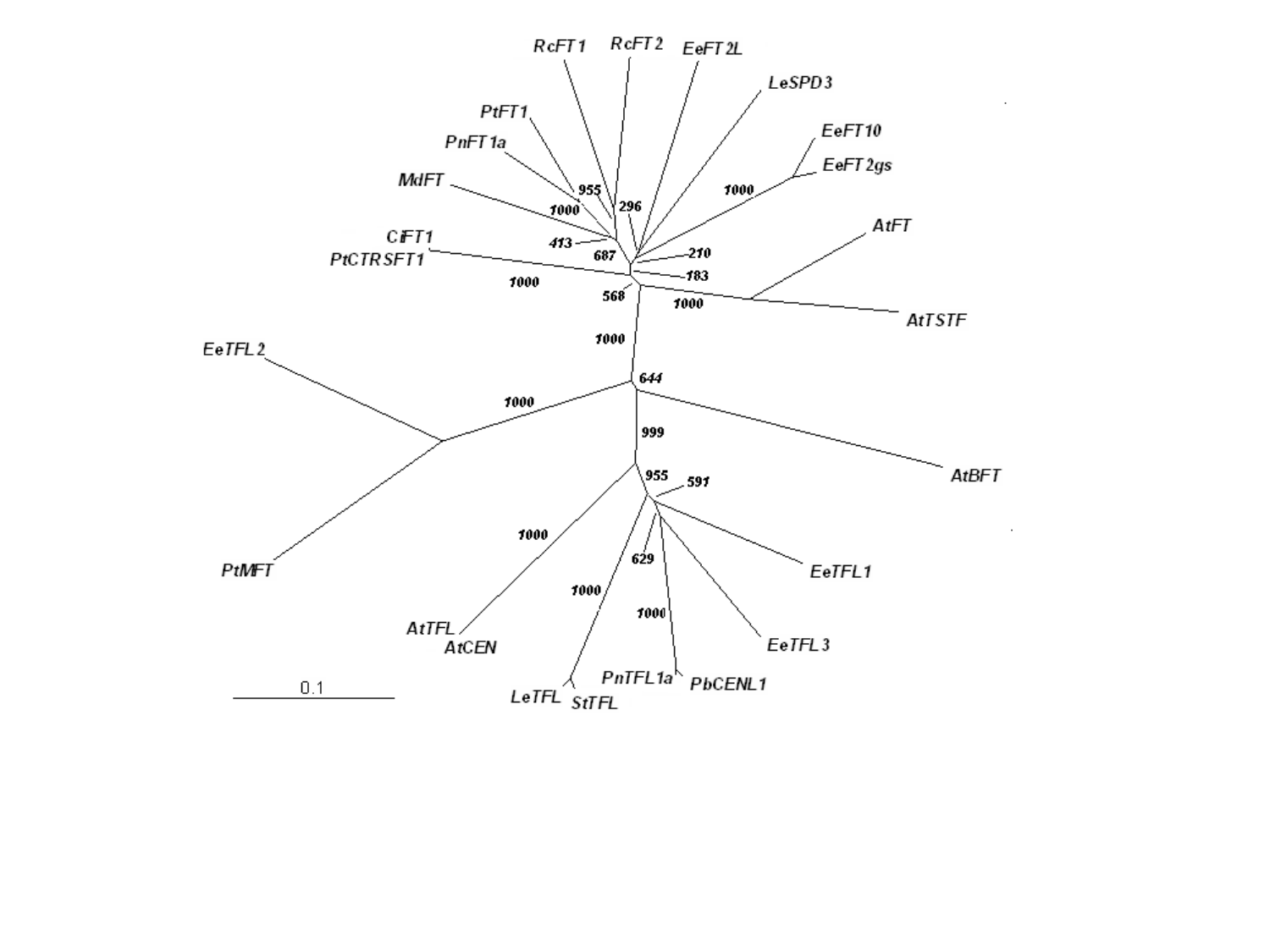

Supplement: Additional file 5 — Cluster analysis of FT and FT-like genes from various species. Phylogenetic analysis of FT-like and TFL-like genes from leafy spurge and various other species. Nucleotide sequence data from four different FT-like genes obtained from leafy spurge (3'Race-EeFT10 (Accession # EU707395), amplification of genomic DNA-EeFTgs (Accession # EU707394, and amplification from cDNA-EeFT2lb (Accession # EU707393) were aligned to several FT-like and TFL-like genes from leafy spurge, poplar, castor bean, Arabidopsis, potato, tomato, and apple using ClustalX. Bootstrap values were generated as shown (1000 iterations). [file 1471-2164-9-536-S5.ppt]

## Slide 1
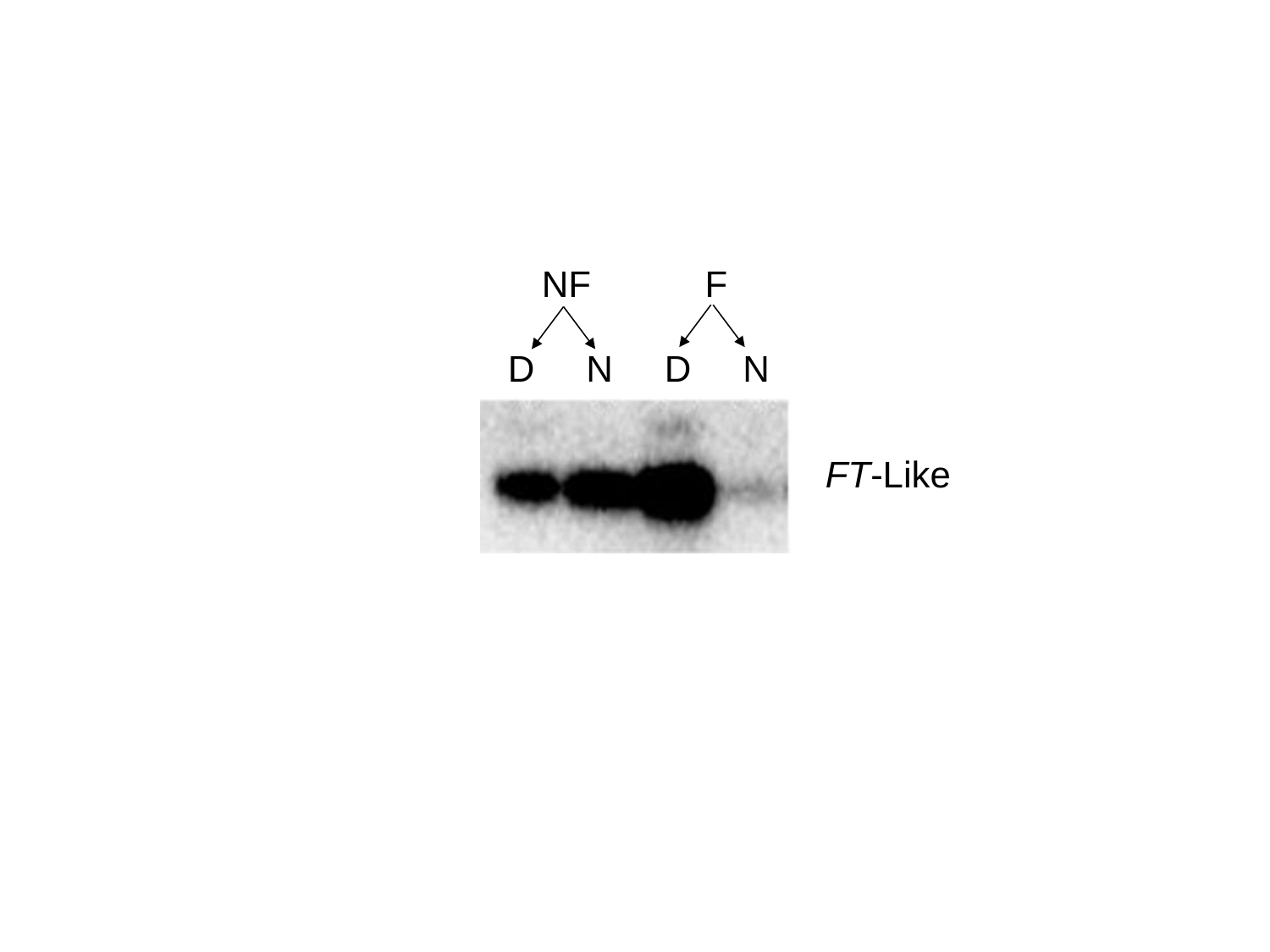

NF F
D N D N
FT-Like

Supplement: Additional file 7 — Northern analysis of FT expression in leafy spurge.Supplemental Figure S3: Northern analysis of RNA collected from leaf tissue approximately 7 hr after dawn (D) and 3 hr after dusk (N) from outdoor-grown flowering competent (F) and greenhouse-grown flowering incompetent (NF) plants. Northern blot was probed with P32 labelled amplified FT-like cDNA fragment (EeFTgb). [file 1471-2164-9-536-S7.ppt]
