# Supplementary material for: Transcriptome analysis identifies novel responses and potential regulatory genes involved in seasonal dormancy transitions of leafy spurge (Euphorbia esula L.)
Source: BMC Genomics. 2008 Nov 12;9:536. doi: 10.1186/1471-2164-9-536 (PMC2605480; doi:10.1186/1471-2164-9-536)
Supplement: Additional file 6 — Sequence data from leafy spurge and Arabidopsis FT genes. Genomic sequence from amplified region of FT-like gene from leafy spurge (top) and equivalent region from genomic DNA sequence of arabidopsis FT gene (bottom). Intron sequence is shown in bold lower case. Identical bases are noted in blue. [file 1471-2164-9-536-S6.ppt]

## Slide 1
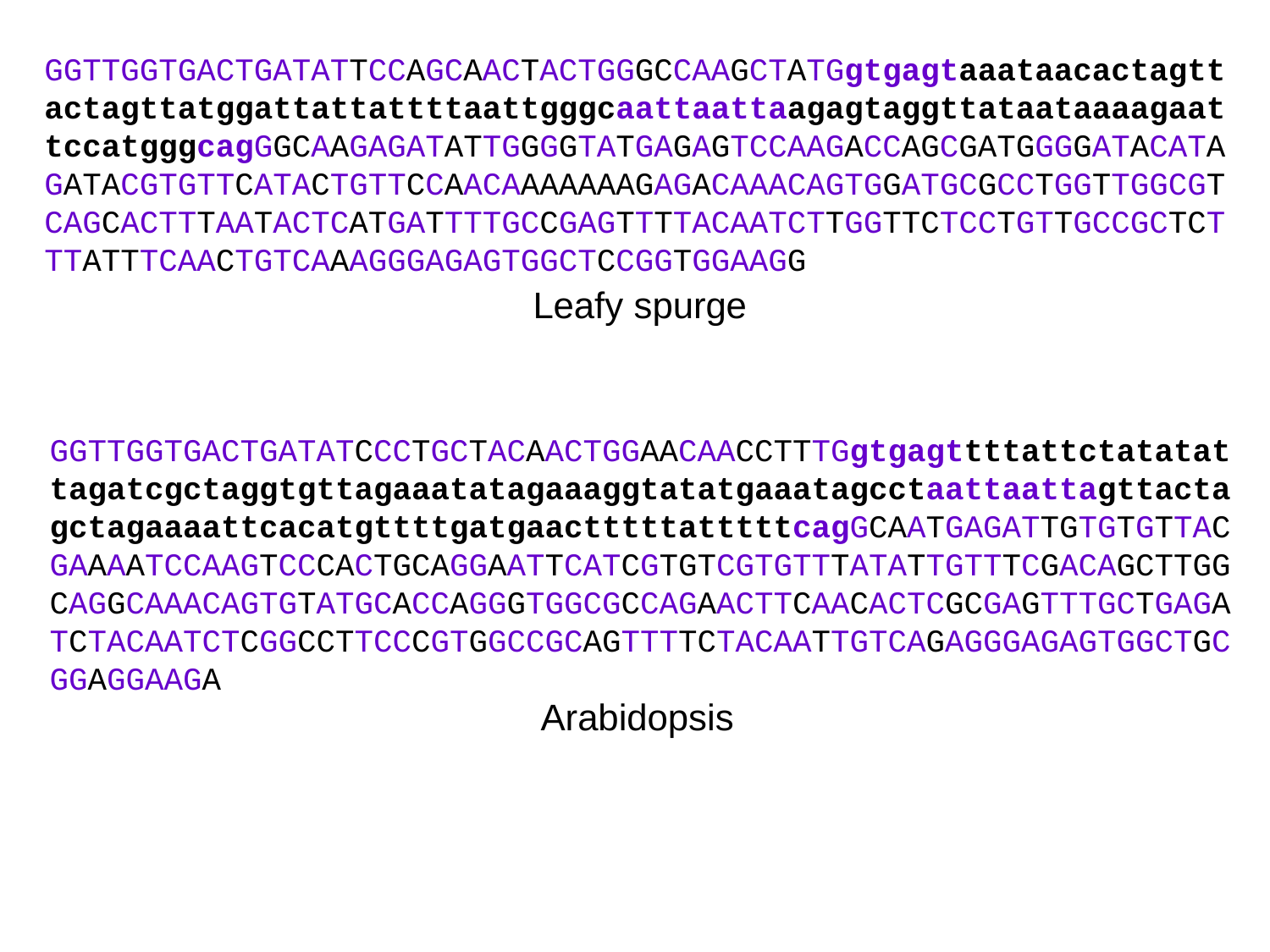

GGTTGGTGACTGATATTCCAGCAACTACTGGGCCAAGCTATGgtgagtaaataacactagttactagttatggattattattttaattgggcaattaattaagagtaggttataataaaagaattccatgggcagGGCAAGAGATATTGGGGTATGAGAGTCCAAGACCAGCGATGGGGATACATAGATACGTGTTCATACTGTTCCAACAAAAAAAGAGACAAACAGTGGATGCGCCTGGTTGGCGTCAGCACTTTAATACTCATGATTTTGCCGAGTTTTACAATCTTGGTTCTCCTGTTGCCGCTCTTTATTTCAACTGTCAAAGGGAGAGTGGCTCCGGTGGAAGG
Leafy spurge
GGTTGGTGACTGATATCCCTGCTACAACTGGAACAACCTTTGgtgagttttattctatatattagatcgctaggtgttagaaatatagaaaggtatatgaaatagcctaattaattagttactagctagaaaattcacatgttttgatgaactttttatttttcagGCAATGAGATTGTGTGTTACGAAAATCCAAGTCCCACTGCAGGAATTCATCGTGTCGTGTTTATATTGTTTCGACAGCTTGGCAGGCAAACAGTGTATGCACCAGGGTGGCGCCAGAACTTCAACACTCGCGAGTTTGCTGAGATCTACAATCTCGGCCTTCCCGTGGCCGCAGTTTTCTACAATTGTCAGAGGGAGAGTGGCTGCGGAGGAAGA
Arabidopsis
